# The Therapeutic Process in Spiritual Healing: Qualitative Results of a Prospective Case Series

**DOI:** 10.1159/000528688

**Published:** 2022-12-15

**Authors:** Barbara Stöckigt, Florian Besch, Claudia M. Witt, Michael Teut

**Affiliations:** Institute for Social Medicine, Epidemiology and Health Economics, Charité Universitätsmedizin Berlin, Berlin, Germany

**Keywords:** Therapeutic process, Therapeutic relationship, Spirituality, Healer, Empathy, Resource, Qualitative research, therapeutischer Prozess, therapeutische Beziehung, Spiritualität, Heiler, Empathie, Ressource, qualitative Forschung

## Abstract

**Introduction:**

The aim of this study was to explore the therapeutic process between contemporary spiritual healers and their clients in Germany.

**Methods:**

This prospective observational case study was supported by questionnaires and semi-structured interviews with clients and healers from the first encounter through a period of 6 months. The qualitative analysis is based on a directed content analysis with focus in this article on the results relating to the therapeutic process.

**Results:**

Seven healers and seven clients were included, and 22 interviews with healers and 20 interviews with clients were conducted. The first treatment session was perceived as laying a foundation for the therapeutic process and the relationship, which was seen as crucial for healing to take place. Healers perceived the therapeutic process as highly individualized and multi-layered, with the perceived effects of spiritual healing sessions layered upon each other. The capacities to connect and to trust were seen as key elements of the healing process. Trust and connection operate multidimensionally: to oneself, to others, and to a transcendent or spiritual source. Clients' spiritual attitudes were regarded as fundamental resources.

**Conclusion:**

The therapeutic process between spiritual healers and their clients was understood as a dynamic and individual activity, building up on a trustful relationship from the first encounter.

## Zusammenfassung

**Hintergrund:**

Ziel dieser Studie war es, den therapeutischen Prozess zwischen zeitgenössischen spirituellen Heilern und ihren Klienten in Deutschland zu untersuchen.

**Methoden:**

In dieser prospektiven Fallstudie wurden Fragebögen und semi-strukturierte Interviews mit Klienten und Heilern von der ersten Begegnung und über einen Zeitraum von sechs Monaten erhoben. Die qualitative Analyse basiert auf einer qualitativen Inhaltsanalyse, wobei der Schwerpunkt in diesem Artikel auf den Ergebnissen zum therapeutischen Prozess liegt.

**Ergebnisse:**

Es wurden sieben Heiler und sieben Klienten rekrutiert und insgesamt 22 Interviews mit Heilern und 20 Interviews mit Klienten geführt. Die erste Behandlungssitzung wurde als Grundsteinlegung für den therapeutischen Prozess und die Beziehung empfunden, die als entscheidend für die Heilung angesehen wurde. Die Heiler nahmen den therapeutischen Prozess als hochgradig individuell und vielschichtig wahr, wobei die wahrgenommenen Wirkungen der spirituellen Heilsitzungen aufeinander aufbauten. Die Fähigkeit, sich zu verbinden und zu vertrauen, wurde als Schlüsselelement des Heilungsprozesses angesehen. Vertrauen und Verbindung wirken multidimensional: zu sich selbst, zu anderen und zu einer transzendenten oder spirituellen Quelle. Die spirituelle Einstellung der Klienten wurde als grundlegende Ressource betrachtet.

**Schlussfolgerung:**

Der therapeutische Prozess zwischen spirituellen Heilern und ihren Klienten wurde dynamisch und individuell verstanden, der von der ersten Begegnung an auf einer vertrauensvollen Beziehung aufbaut.

## Introduction

In Germany, a “healer,” often also called a “spiritual healer,” offers a broad spectrum of healing methods. Some of these techniques are based on traditions, often grounded in religion or even pre-religion; other techniques migrated to Germany due to globalization and as products of the “New Age” [1, 2, 3]. Referring to former publications [4, 5, 6], we define a spiritual healer as a person who works mostly with the laying on of hands, prayer, and/or meditation and, most importantly, considers himself to be able to connect to a spiritual and transcendent source [7, 8]. Transcendence is defined as an experience beyond one's own self, whether linked to spirituality or religion or not [9, 10]. We understand spirituality as a search for the connection to the transcendent, referring to supernatural beings or entities, the divine, or superhuman agents (e.g., God, spirits, angels, the higher Self, a higher power or energy) [11, 12, 13].

Little research exists about the therapeutic process in contemporary spiritual healing in Germany. In psychotherapy, research about theories of change, which began decades ago, aims to investigate complex therapeutic actions, interactions, and changes and is seen as a methodological challenge. Different types of outcomes (immediate, intermediate, and final) should be considered. Different levels of process – the therapeutic relationship, the clients' involvement and contribution in the therapeutic process, significant events and skilful intervention during the therapy, changes in interpretations, and understandings of the client/patient – are seen as important factors [14, 15, 16, 17, 18].

In the field of medical anthropological research, Csordas and Kleinman [19] described the therapeutic process in general as part of a life-long healing process, including the whole domain of the active response to illness, which encompasses all actions that take place, including treatment events, which can consist of various treatment options such as consulting a healer. The therapeutic process also encompasses all intrapsychic phenomena, e.g., the understanding and meaning of the illness, interpretations, and insights. Furthermore, the therapeutic process is embedded in and influenced by the cultural and social context [19].

Within this study, a therapeutic process is understood as all phenomena following a healer's therapeutic interventions, which we observed over a period of 6 months. The therapeutic process is initiated with the client's decision to consult the healer. The therapeutic process does not end with the end of the study. The therapeutic process is separate from the treatment process, which we defined in this study as the process of all treatment sessions included in the study [19]. And finally, the healing process is defined as a personal development process of a client, which does not have to be connected to one specific healer or therapy and can be lifelong [19].

Building on the findings of our former qualitative cross-sectional study [4, 5, 6], this qualitative study investigated changes in the therapeutic process from the first encounter through a period of 6 months. We wanted to learn from healers and their clients about the treatment experiences, the relationships between healers and clients, and the therapeutic process. In the analysis presented in this paper, we focus on the qualitative results of the study related to the therapeutic process.

## Methods

### Design

We used an exploratory observational prospective case series design and applied a mixed-methods approach. In the qualitative part, semi-structured interviews were conducted with healers and clients who had consulted the healer for the first time. Individualized interviews are most suitable in this study because they allow very personal and situative information. The healer and the client were followed for a period of 6 months, with interviews after the first treatment (interview 1), after 2 months (interview 2), and after 6 months (interview 3). Main topics in the interviews for healers and clients were their experiences in the treatment sessions, the perception of the relationship to the healer, respectively, to the client, and their perception of the healing process. Clients were additionally asked about their expectations, possible spiritual experiences during the treatment sessions, and perception of the questionnaires. If possible, participatory observation of treatment sessions was also conducted to understand more about interaction and behaviour. Between the interviews, healers and clients were invited to keep a diary about the therapeutic process.

Because the quantitative part of the study is not related to the therapeutic process, it is not described in detail in this paper. The measurement tools (in the form of validated questionnaires), data collection, data analysis, quantitative results, and qualitative results about perceived short- and long-term effects are all presented in our paper on perceived outcomes [20]. The interdisciplinary research team consisted of three medical doctors and one medical anthropologist.

### Sample

A snowball sampling technique was used to recruit healers [21]. This technique is applicable for populations which are usually difficult to access. As a first step, healers known from our first study were asked to participate. If they elected not to participate, we asked them to recommend other healers. We also searched for healers via the internet (research terms were *spiritual healing*, *spiritual healer*). The participating healers were requested to ask new clients if they were interested in our study. The interested clients were then contacted by the research team by telephone and asked to participate in this study. Each participant signed an informed consent form, and an expense allowance of 100 Euro was paid to each of the healers and the clients. Otherwise, additional treatment costs had to be paid out of the pocket.

Inclusion criteria for healers and clients were 18 years of age and older and written informed consent. Clients should have had no previous treatment by their new healer. Exclusion criteria for healers and clients were lack of knowledge of the German language and also for clients suffering from diseases that would not allow them participating in the study (e.g., cognitive impairment).

### Data Collection and Analysis

All interviews were digitally recorded, transcribed verbatim, and pseudonymized. Written memos of the interviews by the researchers added further information on the setting and the researchers' subjective experiences. The interviews were analysed based on a directed qualitative content analysis with MAXQDA® software [22]. Categories and codes were developed inductively from the data and deductively according to the themes of the structured interview guide and the research question. The analytic process was circular, meaning that new insights from the first data analysis were included in the subsequent data gathering and analysis. The research team met about every 4 months to improve quality and validity of the analysis and to ensure multidisciplinarity and intersubjectivity. All coded interviews were reviewed and re-coded by a randomly assigned team researcher, and results were compared. In a case of disagreement, discussions ensued to reach consensus for further analysis.

## Results

### Recruitment and Sample

In total, 22 healers were asked to participate. Of those, 7 healers were included; 2 healers from our former study and 5 new healers. Reasons for not participating were not working as a healer at the moment, only few clients, change of healing methods, heavy workload, or personal and family reasons. Reasons related to the study design were mistrust in the questionnaires and baseline assessment before any treatment session.

The recruited healers asked 10 clients to participate; 7 clients agreed. Reasons for not participating were mistrust in the questionnaires and cancellation of the first treatment session. Altogether, the recruitment process lasted over 6 months.

The healers were 4 women and 3 men (mean age 55.3 ± SD 5.9 years). Two were “Heilpraktiker” (non-medical complementary and alternative medicine practitioners), one medical doctor, one social worker, one dental technician, one seamstress, and one businesswoman. The clients were all women (mean age 53.1 ± SD 4.9 years). Five were employees, one was a housewife, and one was retired. Reasons to consult a healer were physical complaints (4 with pain, 2 with osteoarthritis, 2 with back pain), psychological problems (2 with depression, 2 with anxiety disorders), and social problems (two clients). In total, 38 interviews with clients and healers were conducted, in a few cases also with healers before the first treatment with the included clients (interview 0). The interviews took place at the healers' or clients' place or at the Charité University. Furthermore, four participatory observations were carried out, and one self-experience of a healing treatment took place. The main and subcategories of the analysis in regard to the therapeutic process are listed in Table 1 and will be described in the following chapters. Detailed information about healing methods, perceptions, expectations, and outcomes are addressed in other publications of our study [4, 5, 6, 20].

### Treatment Process and Procedure

None of the clients said that their therapeutic process had been completed after the 6-month study period. Each considered consulting her healer again if new problems should arise. Five clients ended their treatment process within the first 2 months, during which time, they received from one to five sessions. Two clients received healing sessions throughout the complete 6 months and wanted to continue. C_H6_K1 received one session from a healer in the study and continued with another healer who did not participate in this study (see details in Table 2).

The treatment sessions generally followed a basic structure. Sessions opened with an interview to identify current concerns and the aims of treatment. Three healers reported that beside verbal communication, they intuitively received information from the spiritual world about their client's problems. Those healers reported that they worked with their “*spiritual allies*” and/or saw themselves as mediums and “*channels for healing energy*” by connecting to a universal energy or spiritual source.

After the initial interview, the healing treatment itself was applied. While all healers reported that their work involved the laying on of hands, they also used other spiritual healing methods according to the needs of the client, e.g., aura healing, energy work, soul retrieval, contacts with the “*other world*.” The treatment often ended in silence so that the client could allow himself to linger for a while with the sensations/feelings the session had evoked, see in detail in the paper outcome [20]. Subsequently, a debriefing of the healing session was offered to answer questions or share experiences, which occurred during the treatment.

### D_H2_K2, Client (Interview 2)

“The procedure did not change over time (…) and that is very pleasant. I arrive there (at the healer's place) and first we sit and talk (…) how I am and how we should proceed. Then the treatment takes place. We let it end quietly without having to arrange anything. Then I go. And I like it that way, because it is really allowed to sink in.”

### The Therapeutic Process

#### First Session as Foundation

According to the healers, the first session was of high importance because the “*foundation*” for the whole therapeutic process was laid. The healers reported that especially during the very first session, a quick and – for the clients – impressive improvement often occurred. This improvement was said to be often only short term but could be built upon and strengthened in following sessions. In these first encounters, clients and healers could also explore whether they wanted to work together in the future (see the chapter The Healer-Client Relationship).

### D_H2, Healer (Interview 0)

“There are people who really open up during the first session. They feel unburdened and feel better overall and they are able to hold on to that feeling. (…) They don't have any pain for two or three days. Then it (the pain) returns, but not as bad. They consult me then several times, because the body doesn't really keep up with the soul, I guess. They open up first, let go, then sometimes see things differently, rethink things.”

### Healing Needs Time – the Layers of Healing

Healing was seen by the healers as a lifelong process, which is supported by the healer in the therapeutic process for a certain time. According to most healers in general, a series of about three to four treatment sessions was necessary to see a longer lasting therapeutic success, mainly because chronic problems could not be solved or improved in a sustainable way in only one session. The treatment sessions should ideally build on each other and in each session, only one important issue, e.g., an health complaint, should be addressed. The healing work of one session should afterwards ideally “*sink in*,” which would mean that the new information could be “*integrated*” by the client. In the following session, another issue or complaint could then be addressed. Thus, healer A_H11 explained that even if she could “*solve the problem immediately*,” such a sudden and extreme change could be “*too much*” for the client. The whole system of the client would have to accept and slowly adapt to the changes started by the healing treatments, which could be perceived as new—in some cases scary—and could cause psychological destabilization and also resistance in the client (see also previous quote of healer D_H2). Healer A_H13 used the metaphor of the “*onion*” to explain this process, meaning that healing would occur in one layer after the other, from outside to inside, from the superficial (in the sense of acute and obvious) to deeper—often hidden—layers, and problems. Therefore, most healers expressed the wish to treat the client in a continuous series, thus healing one layer after the other without overloading the client. This conceptualization of the healing process was not always, what the client had in mind. Some clients might already be satisfied about the improvement of acute or more “*superficial*” complaints and might not want to deal with their “*deeper*” problems, e.g., of a socio-psychological nature. Healer A_H9 explained this metaphor of the onion in the therapeutic process as certain “*patterns*” which were built over a long time especially in the case of chronic psychological problems. In such cases, problematic thoughts and behaviour patterns would recur over time in the life of the client, interfering with and blocking a healing process. It would be up to the client to really want to address these patterns and consequently allow enduring healing to happen. This process of letting go or changing a pattern would not be linear, rational, or even conscious, but rather complex and influenced by social, emotional, psychological, and spiritual factors.

The treatment frequency was said to be individually determined. According to the healer, sometimes it makes sense to work more frequently and intensely in the beginning, e.g., every week. As well, breaks of 2–3 weeks seemed to be adequate for integration. However, longer breaks (months or even years) between the sessions might occur. In the end, it is the client who decides, either aligned with the recommendations of the healer or not, because he/she is the one who must be able and willing to take the next step in the healing process.

### A_H13, Healer (Interview 3)

“It is like an onion. (…) You put something down, work on it a little, get healed. Then you get to the next layer and perhaps even to a few more layers during that time. (…) That's 3 weeks or so, although it can sometimes take longer than three weeks. (…) One just has to be ready to take the next step.”

### Ending the Treatment Process

Clients and healers agreed that ending the treatment process would not mean an end to the therapeutic process. All clients except one considered consulting the healer again and all healers said they were agreeable to treating the clients again. Client C_H6_K1 had previously been in contact with another healer and returned to her after she felt uncomfortable with healer C_H6. One client (D_H2_K3) reported that she ended the treatment process because even after five sessions she did not feel any reduction of her symptoms. Three clients reported to have ended their treatments because their symptoms had disappeared or been drastically reduced. Three clients ended the treatment process because they were not able or willing to pay for further sessions (see also Table 2). Although the healers reported that clients' decisions to end treatment did not always align with their recommendations, they nonetheless always accepted the decision of the client without trying to influence such decisions. Some clients shared their decision, some not. Healers sometimes tried to follow-up with the clients who left treatment without explanation. The healers saw their task in “*letting go*” of the client and trusting that clients would find their way back if necessary. In some cases, the clients would resume sessions even after years passed.

### A_H13, Healer (Interview 2)

“I trust that she (client A_H13_K1) would get in contact (…) if something (e.g. a problem) would arise. (…) I do not want to tie anybody to my work (…) I wished (that she) would have come to further treatments (…) and I think that it (the healing process) always would move a little further. But (…) of course, everybody decides on his/her own.”

### The Healer-Client Relationship

#### Establishing Trust

As mentioned before, the healers emphasized the importance of the first contact between the healer and client as establishing a foundation for the further therapeutic process and the relationship. Most clients said that they immediately felt good rapport with the healers; the contact was always “*friendly*,” sometimes even “*amicable*.” This friendly approach would help the clients to trust and to open up. The expression “*to be in good hands*” was used frequently by the clients, see also [5].

The trust-building process was characterized as interplay between the healer and client: the healer offers his healing abilities and empathy, the client, his willingness and openness to the therapeutic process. One-sided trust leads nowhere; the client and healer have to be “*on the same wavelength*” and willing to work together. For the healers, furthermore, trust in a higher, transcendent source plays an important role in their healing work and their relationships with their clients.

### A_H11, Healer (Interview 2)

“That's just teamwork. I have the tools and the people bring the openness with them. (…) They just have a high level of trust in me, right from the start.”

### A_H9, Healer (Interview 1)

“(You've got to) take your client seriously and adjust your thinking to understand that there's a divine order to everything (…) that's our (healers') own trust in the process.”

This is exemplified with a negative example by the case of client C_H6_K1, who ended the therapeutic process with C_H6 immediately after the first encounter because she did not feel comfortable with him.

### Creating a Solid Therapeutic Alliance

In the following interactions between healers and clients, the therapeutic relationship would be strengthened and deepened, wherein the healers' empathy, the healing experiences, deepened trust (especially from the clients' side) and mutual respect would be of high relevance. Over time, the clients reported an increase of trust mainly because of the healing experiences they had during the sessions and the following impacts on their lives (see paper outcome [20]). Clients reported that they were significantly impressed by the, often, charismatic personalities of the healers, their empathetic attitudes, and their healing abilities during treatment sessions. Respect and even admiration for the healer would increase. Healers mentioned respect as a crucial aspect of the relationship, e.g., respect for the willingness of the clients to face their problems.

### A_H9_H10_K1, Client

#### Interview 1

“(The healer is) very empathetic (…) and quite trustworthy.”

#### Interview 3

“And that's why I'm also extremely satisfied with the treatment and with what I experienced (…) And (…) I also just (…) know (…) that there are really people (spiritual healer) who (…) ”see“ more than me as an average person. (…) And yes, that there are really people with a stunning ability.”

All participants agreed that a healer's professional empathetic attitudes are critical for the therapeutic process. The healers often described their contacts with, and approaches to, the clients originating from a place in “*their heart*.” Due to this close, caring relationship, which would grow even more over time, a high level of professionalism and self-reflection in the healers was regarded as important. As a personal friendship had the potential to interfere with the therapeutic process, healers highlighted professionalism and professional distance in their relationships with clients. Professionalism and distance, however, was not considered a significant impediment to intense and empathetic approaches to clients.

### D_H2, Healer (Interview 2)

“For me, these people (the clients) are very close to my heart. It doesn't matter whether or not they want to return, because it's a form of deep, “heart-to-heart,” connection.”

### A_H9, Healer (Interview 2)

“It's not so easy for us. Because we're (…) in quite intimate contact with the person, very personal contact, and we look into (that person's) abyss and they have to be open to that. So, to distinguish exactly which part is the professional part and which the personal part, well, (…) you have to pay attention that you don't mix the two.”

### Spirituality and the Therapeutic Process

Four clients said that already before consulting the healer, spirituality was important for them because it supported them in their life-long healing processes and enriched their lives. During the therapeutic process, they said to experience a confirmation, intensification, and reinforcement of their spirituality, because spiritual healing methods (e.g., laying on of hands, prayer) would offer a tangible spiritual experience.

Thus, in the sense of the threefold relationship (client, healer, the transcendent), the healer supported the client to build or strengthen a transcendent relationship and/or experience. Client A_H9_H10_K1, e.g., saw herself as a rational person with an interest in spirituality, but also with some insecurity and skepticism. She reported that her interests and curiosity, and primarily her suffering and despair, led her to the spiritual treatment. She said to be very impressed by her treatments and to experience a reinforcement of her spiritual interest. She felt “*enriched*” by these experiences and she felt that she had been empowered to gather new and broader perspectives for her life (see also the quote of the client A_H9_H10_K1 in the previous chapter). Client A_H13_K1 even said that spirituality and spiritual healing had saved her life.

### A_H13_K1, Client (Interview 3)

“Spiritual healing (…) and mainly, spirituality, (…) since I decided to go down this path, the more I allow myself to embrace this thinking, the better it goes for me. And consequently, I'm getting stronger. I find that fascinating. (…) Belief-whatever I believe in- is important. For me, in any case. Otherwise, I'd have “taken the bridge” three times by now.”

Three clients said to have a kind of spiritual attitude, e.g., practicing yoga, but this practice would not have any importance for them personally or for their spiritual healing. But even client D_H2_K3, who reported to have no spiritual or religious interest or experiences, imagined that spiritual healing might help her.

### D_H2_K3, Client (Interview 2)

“I can open myself up to this whole issue (spiritual healing). (…) There's (…) actually a wide range of things that can be treated and should be treated and (…) I can imagine, that alongside conventional medicine, this can really help.”

## Discussion

All healers reported using the technique of the laying on of hands while being in contact with transcendent sources. According to the healers, the therapeutic process progressed from outer layers to inner layers, much like peeling an onion. Thus, healers often proposed a treatment series. Sessions were said to build on each other, though did not follow a rational direction or prescribed procedure, but set one step after the other in an intuitive healing process. After establishing a positive initial rapport, the relationship between healers and clients would intensify and trust in one another would deepen. For healers, the first treatment session was seen as the foundation for the whole therapeutic process and the relationship to the client. The relationship was seen as a crucial factor for all participants, in contrast to spiritual attitudes. Although the connection to a transcendent source was fundamental to all healers' work, only four of the clients experienced spirituality as supportive of their healing processes and three clients regarded their spiritual attitudes as not very relevant.

### Strengths and Limitations of the Study

One limitation could be the baseline data collection: Though we decided not to conduct a baseline interview, we had to assume that even only the introduction of information in a baseline questionnaire might influence the therapeutic process. Because some healers found the notion of a baseline questionnaire incompatible with the concept of spiritual healing, they refused to participate in the study. Thus, that might have had some influence on the type of healer willing to participate in the study.

Nonetheless, we decided to include baseline data collection and quantitative data into the study design. This mixed-methods approach allowed a profound and broad analysis. The naturalistic approach in this study with new clients reduced potential selection and community bias we had to face in our former study [4, 5, 6]. Another strength of this study is the prospective design in which we followed the therapeutic process for a length of 6 months. Furthermore, working in an interdisciplinary team, including medicine and anthropology, allowed various perspectives during the whole research process.

### The Two Main Directions of Movement in a Therapeutic Process

We conclude from our qualitative data the theory of a movement into two main directions during the therapeutic process which we will outline as follows: one direction is layer by layer, from the outside (the obvious complaint) to the inside (the deeper aspects connected to the complaint) – like peeling an onion. This metaphor used by one healer fits well with what most healers described. The other direction is from the bottom to the top, wherein the first encounter of the healer and client is the foundation for the following sessions to build upon. We explain that these two directions interweave during the therapeutic process as follows: The first encounter between the healer and the client is the beginning of the whole therapeutic process. Often, the first treatment session would have significant, but not long-lasting, effects on a client, such as a reduction of symptoms or an increase of feelings of well-being. The healers explained that the first encounter shows the client his self-healing potential. Still, because of a patient's own well-established patterns regarding his health, the effects of the treatment would usually not last. The experiences would have to be integrated into “*the whole system*” of the client. Speaking metaphorically, the first layer of the onion was removed and the outer skin peeled. The next treatment sessions are said to build on this first one, to reassure the client of his healing potential and to handle one issue after the other with empathetic sensitivity and without overloading the client, dropping one layer after the other. According to healers' explanations, we postulate that the therapeutic process moves from the superficial and obvious (e.g., the acute complaint of the client) to the deeper, still invisible, and perhaps even hidden aspects or problems of the client (e.g., unconscious psychological aspects).

After one layer is removed, the next layer becomes visible and can therefore be addressed. This is why the healers often propose a treatment series. Still, it is the client's decision where and when to stop the therapeutic process and how far to go in his or her healing process. Clients' wishes do not always correlate with the healers' offers; the clients are often already satisfied after an improvement of their symptoms. Healers, on the other hand, sometimes offer to dig deep to the roots of problems, which the client might not want to face. It is then the task of the healer to accept and respect the client's decision and wherever he is at that point in the therapeutic process in his healing process.

This multi-layered process from the outside and obvious to the inside can be understood as dynamic and unpredictable since what will become visible after one layer has been removed is not knowable. To achieve healing, the client must be prepared to deal with himself and “*to go inward*.” This might allow a deep and consistent transformation but might be uncomfortable, challenging, and evoke feelings of insecurity and fear. The healer has to help the client through this process by creating a robust foundation. This foundation is mainly built through the positive healing experiences during the session itself, its impact on clients' lives, and the deepened trust in a reliable therapeutic relationship. This robust foundation gives the client stability in the dynamic, unknown process and helps him to engage in the therapeutic process.

### The Relationship between the Healer and Client in the Therapeutic Process

The relationship was seen by all participants as the basis for success in the therapeutic process, which could only prosper with teamwork between the client and healer. The healer offered his healing abilities and his empathy; the client offered his willingness to open up for the therapeutic process. Good rapport from the first encounter was seen as essential. While getting to know the healer and his/her work over time, trust in the healer, his healing abilities, and his personality would increase. The results are consistent with findings of our previous study [5]. Psychotherapy research considers the therapeutic relationship as an essential element of therapy [15, 23, 24]. The concept of the therapeutic alliance emphasizes the collaborative aspects of a therapist-client relationship and its importance for a process of change and the outcomes of therapy [25, 26]. Luborsky proposed that the alliance between the therapist and client develops in two phases. In the first phase, type I alliance, the client's belief in the therapist's ability to help him and the therapist's empathetic approach are central. The second phase, type II alliance, emphasizes the client's investment by taking responsibility in the therapeutic process [27]. These two phases can also be applied to our data.

To create a solid therapeutic relationship, healers saw self-reflection as important. The healers also pointed out the importance of respect for the client and his decisions. Hierarchical and partner-based participatory aspects both can be found in our data and should be considered. On the one hand, a client's increased admiration for the healer can promote his possible influence and even power over the client. On the other hand, a client's responsibility for his own healing process is seen as necessary. The healer's respect for the client might foster a feeling of empowerment and positive reinforcement for the client. Another aspect, which could influence the therapeutic process, is when healers' personal closeness and professional empathy are not well differentiated over time. Similarly, clients have to learn to open up and trust a therapist/healer deeply without becoming simultaneously personally connected and involved. Furthermore, the healers reported that they were continuously asked to let go of their own ideas about the therapeutic process. Concepts and research about attachment patterns and expectations between therapists and clients may influence motivation and the therapeutic alliance and relationship [28, 29]. Even though healers asserted that they act intuitively in the moment, critical self-reflection and awareness of their own possible attachments in the therapeutic relationship are warranted.

In the widely accepted concept of the therapeutic alliance, its main dimensions are the affective bond between the therapist and client and agreement on tasks and goals [25, 26]. The importance of these dimensions overlap with the findings of our study related to the importance of the affective bond. The task of the client and therapist seems to be clarified in our sample (healing abilities of the healer, openness, and responsibility of the client), but healers' comments show that this is not always the case, especially as it relates to the client's responsibility. Furthermore, agreement on goals seems to vary sometimes within our sample in the sense of different expectations of the therapeutic process. This also relates to findings in psychotherapy studies, wherein despite the importance of agreement on the mentioned dimensions, clients' and therapists' views often differ and should be reflected [30].

### Trust and Being Connected

The spiritual attitude – to our surprise and in contrast to findings in our former study, wherein most of the clients were spiritually interested – was less important for the clients than expected [4]. Indeed, 3 clients reported that despite having a general spiritual attitude, they did not connect spirituality with their healing processes. But the other 4 clients suggested that they experienced spirituality as an important support in their respective healing processes. Spirituality was seen as an enrichment in their lives, and they reported to have found spiritual reassurance during the therapeutic process. One explanation could be that in the former study, all clients had consulted their healers for a longer duration, sometimes even for years, which might not be the case for clients without spiritual interests. Nevertheless, all clients, including the ones from the former study, consulted the healers primarily because of their problems and complaints and not because of a spiritual search [6].

More important, and essential in the whole therapeutic process, seems to be the ability to make contact, to open up, to relate, and to trust: the healer and client connect, the healer connects to his intuition and a transcendent source, and the client connects to himself, his social surrounding, and potentially (re-)connects to a transcendent source. Trust is required in that process. The client must trust the healer and his work; the healer must trust the client and his decisions about the therapeutic process; a healer must trust in a transcendent source; both parties must trust in a healing potential, and each must trust in himself. Understanding the capacity to connect might be one of the biggest changes during the therapeutic process for the client. To learn to connect to oneself and to cultivate better self-awareness seems to be essential. These connections, combined with the positive experiences during a treatment session may help clients to reframe their understanding of their complaints and give new meaning to their lives.

With our findings we support, therefore, the importance of meaning for the healing process. In process of change research, this relates to the importance of significant events in therapy (here, the positive experience during the treatment session), overcoming pathogenic beliefs (here, new meaning and reframing), and better self-understanding (here, self-awareness), [31]. Hinton and Kirmayer [32] call it the induction of cognitive and emotional flexibility with various healing practices, which promote resilience and well-being. The findings of our study propose that through emotional change, e.g., trusting, feeling connected, positive experiences during the treatment session, cognitive change in the sense of reframing, and new meaning, can result in active changes in life.

## Conclusions

There seem to be two main directions during the therapeutic process: one is dynamically layer by layer, from the outside to the inside, like peeling an onion. The other is from the bottom to the top, wherein the first encounter of the healer and client is the foundation on which subsequent sessions build. A reliable relationship between the healer and client was described as a crucial factor for a successful therapeutic process. A spiritual attitude might be a resource in the healing process but was not seen as most relevant for the clients. More important was the capacity to connect and to trust oneself, the other, and possibly a transcendent source. This would allow for the creation of new meanings in life.

## Statement of Ethics

The study was approved by the Ethics Committee of the Charité - Universitätsmedizin Berlin (EA1/238/10; July 29, 2013). Written informed consent was provided by all participants before participating in the study and for publication.

## Conflicts of Interest Statement

The authors declare that they have no competing interests.

## Funding Sources

The study was funded by the Goerdt-Stiftung im Stifterverband für die Deutsche Wissenschaft – Deutsches Stiftungszentrum, in Essen, Germany. The funding sources had no role in the design and conduct of the study; collection and management, analysis, and interpretation of the data; or preparation, review, or approval of the manuscript.

## Author Contributions

Study concept and design: Michael Teut, Barbara Stöckigt, Claudia Witt, and Florian Besch. Data management: Barbara Stöckigt. Statistical analysis: Michael Teut. Qualitative data collection and analysis: Barbara Stöckigt, Michael Teut, and Florian Besch. Interpretation of data: Barbara Stöckigt, Michael Teut, Florian Besch, and Claudia Witt. Obtaining funding: Claudia Witt and Michael Teut. Drafting the manuscript: Barbara Stöckigt, Michael Teut, Florian Besch, and Claudia Witt. All the authors read and approved the final manuscript.

## Data Availability Statement

The datasets generated and analysed during the current study are not publicly available due to individual privacy of the participants but are available in parts from the corresponding author on reasonable request.

## Figures and Tables

**Table 1:** Main and subcategories

Main categories	Subcategories
1. Recruitment and sample	

2. Treatment process and procedure	

3. The therapeutic process	3.1. First session as foundation
	
	3.2. Healing needs time – the layers of healing
	
	3.3. Ending the treatment process

4. The healer-client relationship	4.1. First session as foundation
	
	4.2. Creating a solid therapeutic alliance

5. Spirituality and the therapeutic process	

**Table 2:** Therapeutic process

Client	Number of treatment sessions	Time point of ending the treatment process	Reasons for ending the treatment process	Successful treatment	Ending of therapeutic process	Reconsulting the healer
A_H9_H10_K1	3	After 2 months	No further needs, all topics addressed	Yes	No	In case of new problems
A_H11_K1	3	After 1 month	Financial constraints	Partially yes*	No	If there is money for it
A_H13_K1	1	After the first treatment session	Currently no further needs	Partially yes*	No	If further topics need to be addressed
D_H2_K2	5	After 2 months	Financial constraints, improvement of symptoms	Yes	No	If there is money for it
D_H2_K3	5	After 2 months	Financial constraints, no improvement of symptoms	No	No	If there is money for it
C_H4_K1	5	–	–	No (partially for short term)	No	Yes
C_H6_K1	1	After the first treatment session	Bad rapport; change to another healer	?	–	–
	Other healer: 5	–	–	Yes	No	Yes

* Not all problems are resolved, still ups and downs, but much better than before treatments.
